# Hyponatremia and increased risk of dementia: A population-based retrospective cohort study

**DOI:** 10.1371/journal.pone.0178977

**Published:** 2017-06-07

**Authors:** Mu-Chi Chung, Tung-Min Yu, Kuo-Hsiung Shu, Ming-Ju Wu, Chao-Hsiang Chang, Chih-Hsin Muo, Chi-Jung Chung

**Affiliations:** 1 Division of Nephrology, Department of Medicine, Taichung Veterans General Hospital, Taichung, Taiwan; 2 School of Medicine, Chung Shan Medical University, Taichung, Taiwan; 3 Department of Urology, China Medical University and Hospital, Taichung, Taiwan; 4 Department of Medicine, College of Medicine, China Medical University and Hospital, Taichung, Taiwan; 5 Department of Public Health, China Medical University, Taichung, Taiwan; 6 Management Office for Health Data, China Medical University and Hospital, Taichung, Taiwan; 7 Department of Health Risk Management, College of Public Health, China Medical University, Taichung, Taiwan; 8 Department of Medical Research, China Medical University and Hospital, Taichung, Taiwan; Istituto Di Ricerche Farmacologiche Mario Negri, ITALY

## Abstract

Hyponatremia is the most common electrolyte disorder and also a predictor of mild cognition impairment. However, the association between hyponatremia and dementia in long follow up periods is rarely investigated. A retrospective cohort study was performed using the claims data of all insured residents who were covered by Taiwan’s universal health insurance from 2000 to 2011. A total of 4900 hyponatremia patients and 19545 matched comparisons were recruited for the analysis. The incidences of hyponatremia and dementia were diagnosed with clinical protocol and defined using the International Classification of Diseases, 9th Revision, Clinical Modification (ICD-9-CM). Cox proportional hazard regression and Kaplan–Meier curves were used for the analyses. Independent of adjusting factors, hyponatremia patients had 2.36-fold higher chances of suffering dementia, including Alzheimer’s disease (AD) and non-AD dementia, than the comparisons. Severe hyponatremia patients had higher risks of suffering dementia than the non-severe hyponatremia patients (adjusted hazard ratio: 4.29 (95% CI: 3.47–5.31) versus 2.08 (95% CI: 1.83–2.37)). A dose response relationship was observed between hyponatremia and dementia. Those hyponatremia patients with baseline or incident stroke had significantly higher chances of suffering dementia compared with those patients without hyponatremia and stroke. Stroke is a significant modifier of the relationship between hyponatremia and dementia. Cerebrovascular disease after incident hyponatremia must be prevented to reduce the incidence of dementia.

## Introduction

Hyponatremia is not only the most common electrolyte disorder but is also associated with higher infection rate [1], cardiovascular disease [2, 3], and mortality risk [4, 5]. Central nervous system (CNS) symptoms are the main manifestations of hyponatremia [6]. Symptoms of acute hyponatremia include confusion, coma, and seizure that is triggered through cerebral edema. In chronic hyponatremia, the degree of cerebral edema becomes mild through osmotic equilibrium, thereby reducing the severity of CNS symptoms. Several studies reported that chronic hyponatremia resulted in mild cognition impairment (MCI) [7, 8], which in turn was associated with increased risk of progression to dementia [9] or death [10]. However, the MCI among patients with hyponatremia was transient and would return to its normal state when the hyponatremia was corrected [8, 11].

Dementia is a progressive, incurable disease that greatly influences the quality of life of the affected patients. About 35.6 million individuals worldwide are suffering from this disease, and this number is expected to triple in value by 2050[12]. Dementia requires a higher medical cost than cancer and heart disease combined[13]. Therefore, many studies attempted to explore the modifiable risk factors of dementia, such as diabetes mellitus (DM), hypertension, and hyperlipidemia [14]. However, hyponatremia, as a clear cause of MCI, is not well-documented for dementia[15]. In fact, cerebrovascular diseases are currently considered as underlying pathologic hallmarks of various types of dementias[16]. Hyponatremia has also been suggested an important predictor of cerebrovascular diseases, such as stroke [2, 3]. Therefore, the association between hyponatremia and dementia must be explored further.

This study is the first to explore such association based on a retrospective cohort study from a nationwide database. This study attempts to determine the following: (1) the association between hyponatremia and dementia, including Alzheimer’s disease (AD) and non-AD dementia; (2) the relationship among hyponatremia, stroke, and dementia; and (3) the dose response relationship between hyponatremia severity and dementia.

## Materials and methods

### Data source

The Taiwan Department of Health consolidated 13 insurance programs to establish the Taiwan National Health Insurance program (NHI program) in March 1995. Over 99% of Taiwanese residents are covered by this program. The National Health Insurance Research Database (NHIRD) comprises NHI program registration files and original claims data for reimbursement. This database was established by the National Health Research Institute, which was authorized to manage insurance data. As a sub-dataset of NHIRD, the Longitudinal Health Insurance Database (LHID 2000) includes claims data for one million people who have been randomly selected from the total insured population between 1996 and 2011. We selected our sample from LHID 2000 to investigate the association between hyponatremia and dementia. This database also contained information on the demographic status of the insured individuals and the claims data for inpatient and outpatient care. The personal information of the participants was concealed to protect their privacy. We ensured that all data were de-identified and analyzed anonymously. This study was also exempted from full ethical review by the institutional review board of China Medical University (CMUH104-REC2-115). The diagnosis of each patient was identified using the International Classification of Diseases, 9th Revision, Clinical Modification (ICD-9-CM).

### Study population

Using the retrospective population-based cohort design, we identified 4900 new hyponatremia (ICD-9-CM code 276.1) patients, whose first-time hyponatremia diagnoses were used as index year (2000 to 2009). Given that severe symptomatic hyponatremia patients could be treated with hypertonic saline (3% saline) in clinical settings, we defined the severe hyponatremia group as those hyponatremia patients who required 3% sodium chloride treatment. By contrast, the non-severe hyponatremia group included hyponatremia patients that did not require 3% sodium chloride treatment. For comparison cohort (non-hyponatremia cohort), we randomly selected 19545 subjects without hypernatremia (ICD-9-CM: 276.0) and hyponatremia diagnosis at a 1:4 ratio. These cohorts were frequency-matched by age (per five years), gender, and index year.

### Study variables

The follow-up person–years at the end of 2011 were calculated for each subject until the diagnosis of dementia (ICD-9-CM: 290, 294.1, and 331.0) or withdrawal from the insurance system. We categorized dementia into AD (ICD-9-CM: 331.0) and non-AD (ICD-9-CM: 290 and 294.1). Those subjects with a history of dementia at the baseline were excluded from the study. All dementia-related comorbidities, including diabetes (ICD-9-CM: 250), hypertension (ICD-9-CM: 401 to 405), hyperlipidemia (ICD-9-CM: 272), ischemic heart disease (ICD-9-CM: 410 to 414), heart failure (ICD-9-CM: 428), mental illness (ICD-9-CM: 290 to 319), stroke (ICD-9-CM: 430 to 438), liver cirrhosis (ICD-9-CM: 571.2, 571.5, and 571.6), atrial fibrillation (ICD-9-CM: 427.31), chronic renal disease (ICD-9-CM: 585), parkinsonism (ICD-9-CM: 332), and cancer (ICD-9-CM: 140 to 208), were identified before the index date. Diuretics use was evaluated before the index date and was then divided into furosemide, thiazide, and other diuretics. In addition, we defined the visiting numbers as sum of both outpatient and inpatient visit for hyponatremia per year to be representative of severity of hyponatremia. To explore the interaction effect between hyponatremia and stroke on dementia risk, we defined baseline stroke as the occurrence of stroke event before the index date of hyponatremia and defined incident stroke as the occurrence of stroke event after the index date of hyponatremia.

### Statistical analysis

We performed chi-square and student's t tests to examine the differences among the categorical and continuous variables, respectively. We employed analysis of variance to analyze the differences in the mean age and follow-up years of patients in the severe hyponatremia group, patients in the non-severe hyponatremia group, and comparison cohorts. The incidence rate ratio of dementia was measured via Poisson regression analysis. The multivariable Cox proportional hazards model was used to adjust dementia-related comorbidities and to estimate the risk of dementia, which was represented by adjusted hazard ratio (aHR) and 95% confidence interval (95% CI). Kaplan–Meier analysis was performed to measure the cumulative dementia incidence for the three study groups, and the log-rank test was performed to assess the differences of cumulative incidence among these groups. All statistical analyses were performed using the SAS 9.4 statistical package (SAS Institute Inc., NC, USA), with a significance level of *P* <0.05 in the two-tailed tests.

## Results

**Fig 1:**
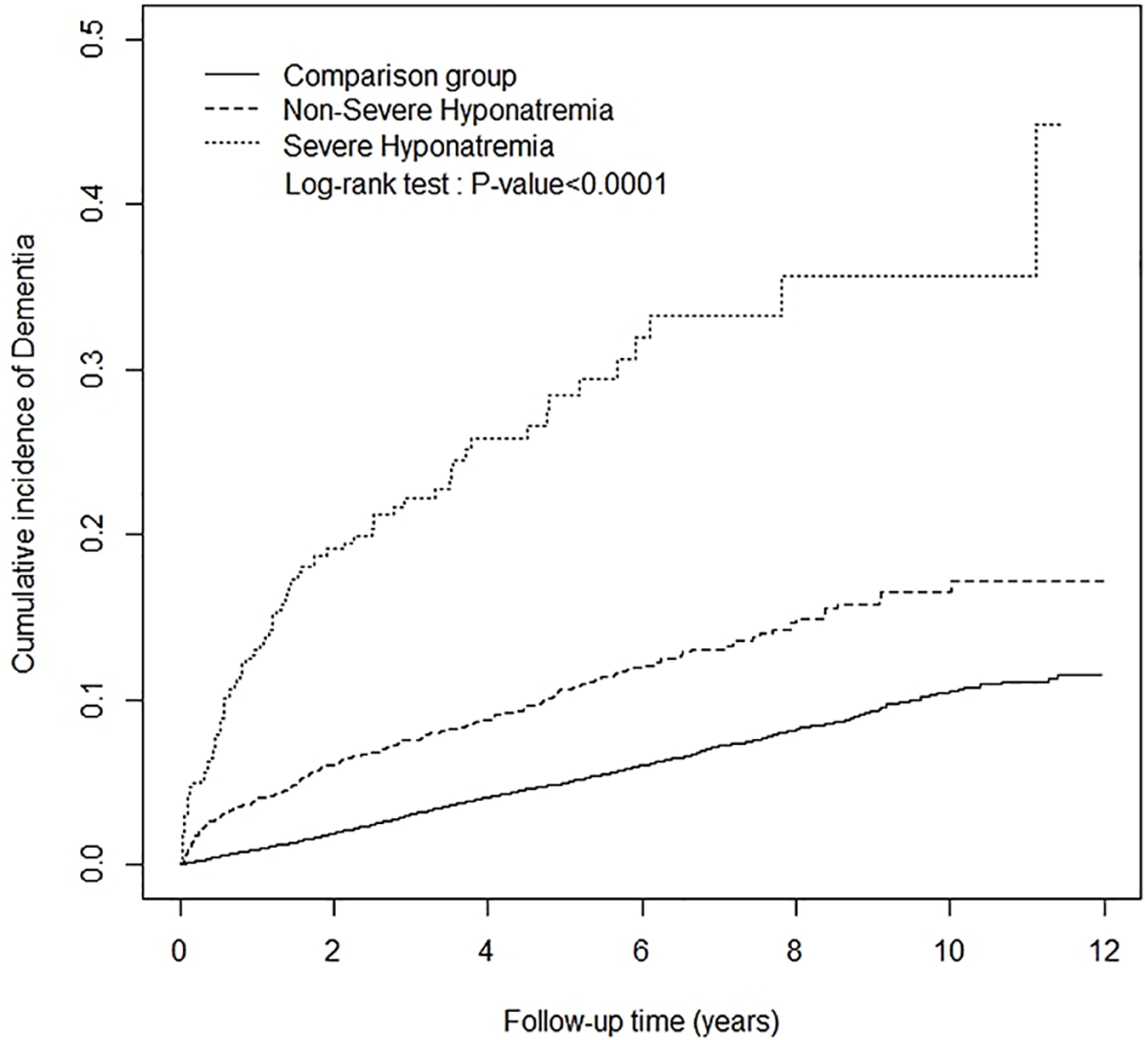
Cumulative dementia risk in the severe hyponatremia, non-severe hyponatremia, and comparison groups.

Table 1 shows the demographic characteristics, status of each comorbidity and uses of diuretics by the hyponatremia (N = 4900) and non-hyponatremia cohorts (N = 19545). The cohorts in both groups had similar age and sex distributions after frequency matching. The mean age in both groups was approximately 67 years. The hyponatremia group had a higher prevalence of comorbidity and percentage of diuretics use than the non-hyponatremia group. We further defined the severe (N = 572) and non-severe hyponatremia groups (N = 4328). The severe and non-severe hyponatremia groups (chi-square test p-value < 0.0001) had different age, sex, comorbidity, and diuretics use distributions than the non-hyponatremia cohort. The mean follow-up years for dementia were 2.16, 3.20, and 5.16 in the severe hyponatremia group, non-severe hypernatremia group, and comparison group, respectively. The severe hyponatremia group showed the highest cumulative incidence of dementia by the end of the follow-up period (Fig 1; Log-rank test p-value < 0.0001).

**Table 1:** Demographic profiles of patients with hyponatremia, divided into severe and non-severe hyponatremia.

	Hyponatremia									
	Severe		Non-Severe		All		Comparison			
	N = 572		N = 4328		N = 4900		N = 19545			
	n	%	n	%	n	%	n	%	p-value^1^	p-value^2^
Age, year									0.89	< 0.0001
< 65	136	23.8	1661	38.4	1797	36.7	7188	36.8		
≥ 65	436	76.2	2667	61.6	3103	63.3	12357	63.2		
Mean (SD)	72.1 (13.5)		66.7 (16.5)		67.1 (16.2)		67.3 (16.3)		0.32^†^	< 0.0001^#^
Gender									0.90	0.94
Women	260	45.5	1935	44.7	2195	44.8	8736	44.7		
Men	312	54.5	2393	55.3	2705	55.2	10809	55.3		
Comorbidity										
Diabetes	237	41.4	1761	40.7	1998	40.8	4206	21.5	< 0.0001	< 0.0001
Hypertension	436	76.2	2918	67.4	3354	68.4	10313	52.8	< 0.0001	< 0.0001
Hyperlipidemia	179	31.3	1432	33.1	1611	32.9	5389	27.6	< 0.0001	< 0.0001
Ischemic heart disease	269	47.0	1699	39.3	1968	40.2	5854	30.0	< 0.0001	< 0.0001
Heart failure	121	21.2	716	16.5	837	17.1	1572	8.04	< 0.0001	< 0.0001
Mental illness	299	52.3	2003	46.3	2302	47.0	6797	34.8	< 0.0001	< 0.0001
Stroke	324	56.6	1621	37.5	1945	39.7	3954	20.2	< 0.0001	< 0.0001
Liver cirrhosis	50	8.74	358	8.27	408	8.33	358	1.83	< 0.0001	< 0.0001
Atrial fibrillation	58	10.1	264	6.10	322	6.57	513	2.62	< 0.0001	< 0.0001
CKD	50	8.74	353	8.16	403	8.22	594	3.04	< 0.0001	< 0.0001
Parkinsonism	47	8.22	222	5.13	269	5.49	417	2.13	< 0.0001	< 0.0001
Cancer										
Brain	3	0.52	9	0.21	12	0.24	3	0.02	< 0.0001	< 0.0001
Non-brain	98	17.1	606	14.0	704	14.4	870	4.45	< 0.0001	< 0.0001
Diuretics										
Furosemide	277	48.4	1701	39.3	1978	40.4	3398	17.4	< 0.0001	< 0.0001
Thiazide	57	10.0	221	5.11	278	5.67	669	3.42	< 0.0001	< 0.0001
Others	57	10.0	244	5.64	301	6.14	400	2.05	< 0.0001	< 0.0001
Follow-up years										
Mean (SD)	2.16 (2.53)		3.20 (2.91)		3.10 (2.89)		5.16 (2.81)		< 0.0001^†^	< 0.0001^#^

SD, standard deviation; Chi-square

^†^t-test

^#^ANOVA. p-value

^1^: the p-value between all hyponatremia and comparison groups. p-value

^2^: the p-value for different types of dysnatremia relative to that for the comparison group

After considering about the same conditions of dementia-related comorbidities, Table 2 shows that the hyponatremia cohort has 2.36-fold higher chances of suffering dementia than the comparison cohort (95% CI: 2.09 to 2.66) using the multivariable Cox proportional hazards model. Compared with the non-hyponatremia cohort, the risk of dementia significantly increased in the severe (aHR: 4.29, 95% CI: 3.47 to 5.31) and non-severe hyponatremia groups (aHR: 2.08, 95% CI: 1.83 to 2.37). In addition, we evaluated the visiting numbers per year for hyponatremia cohorts and further explored the effect of visiting numbers on dementia risk. The risk of dementia increased from 1.54 (95% CI: 1.33 to 1.77) for those patients with two or fewer visiting numbers up to 15.7 (95% CI: 13.1 to 19.0) for those patients with more than two visiting numbers (p-value < 0.0001 for trend). Hyponatremia patients had 2.31- (95% CI: 1.35 to 3.95) and 2.36-fold (95% CI: 2.09 to 2.66) higher chances of suffering AD and non-AD dementia than the non-hyponatremia patients. Non-severe hyponatremia patients had statistically significant higher risks of suffering AD than the comparison cohort (aHR: 2.35, 95% CI: 1.34 to 4.10). However, an insignificant increased risk was observed between severe hyponatremia and AD. Compared with that of the comparison cohort, the risk of non-AD was 4.38 (95% CI: 3.53 to 5.43) and 2.07 (95% CI: 1.81 to 2.36) in the severe and non-severe hyponatremia groups, respectively.

**Table 2:** Incidence rate and hazard ratios of dementia in different severity and visiting numbers of hyponatremia.

Outcome	Event	Person–Years	Rate	Crude HR (95% CI)	Adjusted HR (95% CI) ^#^
Dementia (ICD-9-CM 290, 294.1 and 331.0)					
Comparison cohort	1056	100778	10.5	1.00	1.00
Hyponatremia cohort	424	15081	28.1	2.61(2.33 to 2.93)***	2.36(2.09 to 2.66)***
Non-severe hypernatremia	324	13843	23.4	2.19(1.93 to 2.48)***	2.08(1.83 to 2.37)***
Severe hypernatremia	100	1238	80.8	7.32(5.96 to 9.00)***	4.29(3.47 to 5.31)***
Hyponatremia visiting numbers, per year					
≤ 2	248	14465	17.2	1.64(1.49 to 1.79)***	1.54(1.33 to 1.77)***
> 2	176	616	285.7	27.3(24.6 to 30.3)***	15.7(13.1 to 19.0)***
p-value for trend				< 0.0001	< 0.0001
Alzheimer disease (ICD-9-CM 331.0)					
Comparison cohort	61	100778	0.61	1.00	1.00
Hyponatremia cohort	19	15081	1.26	2.13(1.27 to 3.57)**	2.31(1.35 to 3.95)**
Non-severe hypernatremia	17	13843	1.23	2.07(1.21 to 3.55)**	2.35(1.34 to 4.10)**
Severe hypernatremia	2	1238	1.62	2.81(0.69 to 11.5)	2.04(0.49 to 8.53)
Non-Alzheimer dementia (ICD-9-CM 290, and 294.1)					
Comparison cohort	995	100778	9.87	1.00	1.00
Hyponatremia cohort	405	15081	26.9	2.64(2.35 to 2.97)***	2.36(2.09 to 2.66)***
Non-severe hypernatremia	307	13843	22.2	2.19(1.93 to 2.49)***	2.07(1.81 to 2.36)***
Severe hypernatremia	98	1238	79.2	7.57(6.15 to 9.33)***	4.38(3.53 to 5.43)***

Medical visit, including outpatient and inpatient visits. PY, person–year; Rate, incidence rate (per 1,000 person–years); IRR, incidence rate ratio; ^#^Adjusted for age, gender, comorbidity, and medicine used

** p < 0.01

*** p < 0.001.

**Fig 2:**
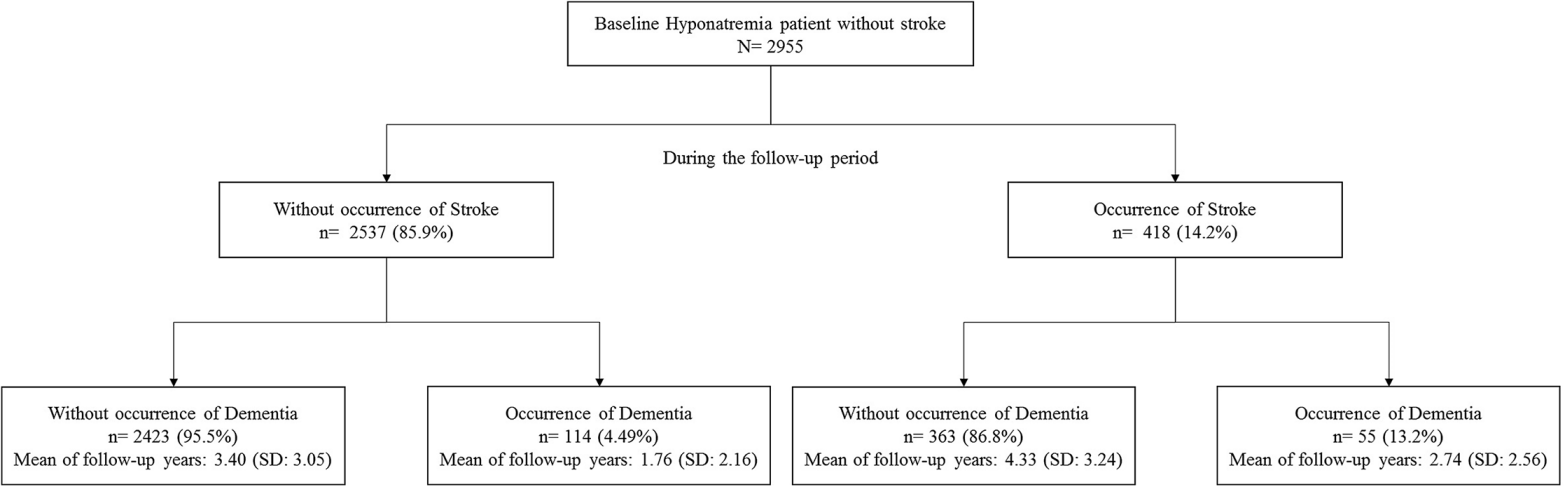
Follow-up occurrence of dementia and incident stroke among hyponatremia patients without baseline stroke.

Table 3 illustrates the joint effect of baseline or incident stroke and hyponatremia on dementia outcomes. Hyponatremia patients with baseline stroke had significantly higher risks of suffering dementia (aHR: 4.63, 95% CI: 4.00 to 5.37) than non-hyponatremia patients without stroke. The association between incident stroke and dementia risk for hyponatremia patients were elucidated during the study period (Fig 2). A total of 2955 hyponatremia patients had no baseline stroke. A total of 418 hyponatremia patients had incident stroke (14.2%) during the follow-up period, among which 55 developed signs of dementia by the end of the follow-up period. A total of 114 patients without incident stroke developed dementia. During the study period, the risk of dementia increased from 1.95 (95% CI: 1.63 to 2.33) for hyponatremia patients without incident stroke to 4.04 (95% CI: 3.05 to 5.34) for hyponatremia patients with incident stroke (Table 3).

**Table 3:** The joint effect between baseline or incident stroke as well as hyponatremia on dementia risk.

Variable		N	Event	PY	Rate	IRR (95% CI)	Adjusted HR^#^	p-value
							(95% CI)	
Hyponatremia	Baseline Stroke							0.7310
No	No	15591	636	83488	7.62	1.00	1.00	
No	Yes	3954	420	17289	24.3	3.19(2.94 to 3.46)***	1.82(1.60 to 2.06)***	
Yes	No	2955	169	10164	16.6	2.18(1.95 to 2.44)***	2.65(2.24 to 3.15)***	
Yes	Yes	1945	255	4917	51.9	6.81(6.19 to 7.49)***	4.63(4.00 to 5.37)***	
Hyponatremia	Incident Stroke							0.1233
No	No	14193	469	74939	6.26	1.00	1.00	
No	Yes	1398	167	8549	19.5	3.12(2.82 to 3.46)***	1.95(1.63to 2.33)***	
Yes	No	2537	114	8441	13.5	2.16(1.92 to 2.43)***	2.77(2.25 to 3.40)***	
Yes	Yes	418	55	1722	31.9	5.10(4.34 to 6.01)***	4.04(3.05 to 5.34)***	

PY, person–year; Rate, incidence rate (per 1,000 person–years); IRR, incidence rate ratio

^#^Adjusted for age and gender; *p*-value for interaction

*** p < 0.001.

## Discussion

To the best of our knowledge, this study is the first nationwide database study to reveal the significant influence of hyponatremia on the occurrence of dementia. Hyponatremia patients had 2.36-fold higher chances of suffering dementia after adjusting other confounding factors. Our findings were consolidated by the effects of the significant dose response relationship between severe and non-severe hyponatremia on dementia occurrence.

Dementia can be divided into different subtypes according to their cause [17]. As the most common subtype, AD accounts for about 50% of all dementia cases. The pathological hallmarks of AD include amyloid plaque and neurofibrillary tangles that damage the structure and function of neurons and synapses. Vascular dementia, which is triggered by a cerebrovascular disease that hinders blood from flowing to the brain, accounts for the majority of the non-AD dementia causes. However, mixed dementia, or the coexistence of AD and non-AD dementia, has been recently identified as the leading cause of dementia [18–20]. The overlap of AD neuropathology (amyloid plaques and neurofibrillary tangles) with cerebrovascular lesions is observed in up to 50% of dementia cases[21]. Therefore, AD and non-AD dementia cannot be easily distinguished in clinical practice. In our study, those patients with non-AD dementia outnumbered those with AD in both normal- and hypo-natremia groups, which indicated that our neurologists tended to classify those patients with signs of mixed dementia to the non-AD group. Regardless of dementia subtype, hyponatremia was significantly associated with higher risks of suffering either AD or non-AD dementia. However, the number of severe hyponatremia patients in our study is too small to show significant results. Dementia has many risk factors[22], including age, sex, DM, hypertension, hyperlipidemia, atrial fibrillation, coronary artery diseases, mental illness, and Parkinson’s disease, which we considered as confounding factors of the disease. Hyponatremia remained an independent strong factor of dementia after these confounding factors were adjusted.

Although we lacked data on the level of serum sodium, we partially compensated for this limitation by investigating the dose response relationship between severe and non-severe hyponatremia. The severity of hyponatremia was measured based on symptoms instead of serum sodium level. All patients with severe symptomatic hyponatremia also received 3% saline in practice[6]. Therefore, our definition of severe hyponatremia was logical. Previous studies suggested that the cognition impairments triggered by hyponatremia could still be treated[8, 9]. However, we found that hyponatremia in long follow-up periods carried a much higher risk of dementia. Therefore, hyponatremia must be prevented to reduce the burden of dementia.

The association between hyponatremia and dementia could be explained in several ways. Hyponatremia could lead to brain damage through multiple mechanisms, predisposing to dementia. Hyponatremia leads to cerebral edema in the acute phase. Cerebral edema was closely linked to neuronal death by the Na/K ATPase dysfunction and the formation of reactive oxygen species[23]. Moreover, *hypoxia* is a major factor in hyponatremia that contributes to brain damage. This condition is usually observed among patients that face hyponatremia encephalopathy through two mechanisms, namely, neurogenic pulmonary edema and hypercapnia respiratory failure.[24] Hypoxia not only deteriorates cerebral edema[25] but also contributes to increased resting blood pressure and cerebral vascular resistance[26]; in addition, hypoxia induces the expression of proinflammatory transcription factors that can lead to endothelial dysfunction, atherosclerosis, and stroke[27].

In chronic hyponatremia, hyponatremia is associated with dementia through multiple mechanisms. First, renin–angiotensin–system (RAS) activation may have a role in the formation of dementia. Hyponatremia serves not only as a marker but also as a contributory factor to RAS activation[28]. Such activation may result in atherosclerosis and lead to plaque rupture in the atherosclerotic lesions of patients with stroke and brain damage[29–31]. Second, hyponatremia is considered a marker of inflammation[32]. Elevated levels of inflammatory cytokines have been observed among hyponatremia patients. Chronic inflammation is also associated with dementia[33]. These cytokines increase the concentration of amyloidogenic peptides, which are central pathogeneses of AD. Third, previous studies showed that hyponatremia could induce mitochondria dysfunction[34] and oxidative stress[35], which also served key pathogenic roles in AD[16]. The abovementioned evidence also suggested the convergence of pathogenic factors on cerebral blood vessels, which could lead to stroke and dementia[20]. Fourth, hippocampus has a major role in triggering dementia. Chronic hyponatremia induces memory loss by decreasing the ATP production and energy of hippocampus cells and by decreasing the long-term potentiation of hippocampal synapses[34]. Fifth, in the animal model, hyponatremia increases the sensitivity of beta amyloid, thereby leading to cognitive impairment[36].

Stroke is notorious for doubling the risk of suffering dementia (post-stroke dementia), including both AD and non-AD dementia[37]. Recurrent or multiple strokes carry much higher risks of dementia[38]. In our study, both baseline and incident stroke increased the risk of dementia, and their interactions with hyponatremia could increase such risk further. These findings indicated the cumulative damage of stroke and hyponatremia. Several risk factors of post-stroke dementia were also identified[38]. Based on our findings, hyponatremia should also be considered when investigating the causes of dementia.

Hyponatremia has a mutual interaction with stroke. Hyponatremia was associated with increased incidence of cerebrovascular diseases [2, 3] and a common complication after cerebrovascular diseases [39]. Fig 2 shows that among those patients without baseline stroke, about 30% (55/169) have developed dementia after suffering a stroke. In conclusion, stroke is an important, yet not essential, modifier on dementia because hyponatremia is an independent risk factor for dementia. Therefore, the abovementioned mechanisms, including vascular or non-vascular factors, must be considered to explain the association between hyponatremia and dementia.

This study has several limitations. First, we had no detailed information about the level of serum sodium, but tried to compensate for this limitation by dividing the sample into severe and non-severe hyponatremia patients. Second, we could not consider all possible confounders of dementia, such as smoking habits, alcohol use, other prescription drugs, vitamin deficiency, education, physical activity, and genetic factors. Third, we could not differentiate the severity and location of stroke, which could also influence the occurrence of dementia.

## Conclusions

In sum, hyponatremia increases the risk of dementia, including both AD and non-AD dementia. Severe hyponatremia carries a much higher risk of dementia. Baseline or incident stroke can modify the relationship between hyponatremia and dementia. To prevent the occurrence of dementia after hyponatremia, clinical physicians must prevent secondary insults, such as cerebrovascular diseases.
